# A novel antigen capture ELISA for the specific detection of IgG antibodies to elephant endotheliotropic herpes virus

**DOI:** 10.1186/s12917-015-0522-6

**Published:** 2015-08-14

**Authors:** Petra B. van den Doel, Víctor Rodríguez Prieto, Sarah E. van Rossum-Fikkert, Willem Schaftenaar, Erin Latimer, Lauren Howard, Sarah Chapman, Nic Masters, Albert D. M. E. Osterhaus, Paul D. Ling, Akbar Dastjerdi, Byron Martina

**Affiliations:** ViroScience Lab, Erasmus Medical Center, Erasmus MC, Room Ee1714, dr. Molewaterplein 50, 3015 GE Rotterdam, The Netherlands; Artemis One Health Research Institute, Utrecht, The Netherlands; VISAVET Centre, Animal Health Department, Complutense University of Madrid, Madrid, Spain; Department of Genetics, Erasmus Medical Center, Rotterdam, The Netherlands; Veterinary services, Rotterdam Zoo, Rotterdam, The Netherlands; Smithsonian Conservation Biology Institute, Smithsonian’s National Zoo, Washington, DC USA; Department of Animal Health, Houston Zoo, Inc., Houston, TX USA; East-Midland Zoological Society, Twycross Zoo, Warwickshire, UK; Veterinary Services, Zoological Society of London, London, UK; Department of Molecular Virology and Microbiology, Baylor’s College of Medicine, Houston, TX USA; Animal and Plant Health Agency, Addlestone, UK

**Keywords:** EEHV, Asian elephant, Glycoprotein B, ELISA, Seroprevalence

## Abstract

**Background:**

Elephants are classified as critically endangered animals by the International Union for Conservation of Species (IUCN). Elephant endotheliotropic herpesvirus (EEHV) poses a large threat to breeding programs of captive Asian elephants by causing fatal haemorrhagic disease. EEHV infection is detected by PCR in samples from both clinically ill and asymptomatic elephants with an active infection, whereas latent carriers can be distinguished exclusively via serological assays. To date, identification of latent carriers has been challenging, since there are no serological assays capable of detecting seropositive elephants.

**Results:**

Here we describe a novel ELISA that specifically detects EEHV antibodies circulating in Asian elephant plasma/serum. Approximately 80 % of PCR positive elephants display EEHV-specific antibodies. Monitoring three Asian elephant herds from European zoos revealed that the serostatus of elephants within a herd varied from non-detectable to high titers. The antibody titers showed typical herpes-like rise-and-fall patterns in time which occur in all seropositive animals in the herd more or less simultaneously.

**Conclusions:**

This study shows that the developed ELISA is suitable to detect antibodies specific to EEHV. It allows study of EEHV seroprevalence in Asian elephants. Results confirm that EEHV prevalence among Asian elephants (whether captive-born or wild-caught) is high.

**Electronic supplementary material:**

The online version of this article (doi:10.1186/s12917-015-0522-6) contains supplementary material, which is available to authorized users.

## Background

The family *Herpesviridae* comprises a diverse group of viruses, which are divided into three subfamilies (*α*-, *β*- and *γ-herpesviridae*) [1]. Most commonly, herpesvirus infection presents as a benign, self-limiting disease with a mild viraemia. Herpesvirus rarely causes widespread disseminated disease in the natural host, although cross-species transmission to non-reservoir hosts can also result in lethal infections [2]. In general neonates and immune-compromised hosts are at higher risk of developing a fatal herpesvirus infection [3, 4].

The elephant endotheliotropic herpesvirus (EEHV) is a poorly characterized virus of elephants that was first observed in healthy wild African elephants (*Loxodonta africana*) by McCully et al. [5] in 1971 and subsequently in Asian elephants (*Elephas maximus*) with no apparent disease [6]. In 1990 the death of a 3-year old female Asian circus elephant was associated with herpesvirus by Ossent et al. [7]. Pathologic changes in the capillary endothelial cells with herpetic inclusion bodies and weak cross-neutralization in serum neutralization tests with bovine herpesviruses suggested that the etiologic agent was a hitherto unidentified herpesvirus [8, 9], which was identified in 1999 [10]. The disease has a sudden onset characterized by lethargy, oral ulcerations, edema in the skin of the head and proboscis, cyanosis of the tongue, decreased white blood cell and platelet count and massive internal hemorrhages, which lead to death within 12–72 h after onset of the symptoms. Phylogenetic analysis showed that EEHV is a distinct branch within the β-herpesviridae group, assigned as the new genus *Proboscivirus* [11, 12]. To date, as many as seven different genotypes of EEHV have been identified within this genus [13]. However, xtensive evaluation of several subtypes indicated that EEHVs have a large genomic inversion of a 40-kb core segment that is distinct from the Roseoloviruses and all other β-herpesviruses. Furthermore, they encode α-herpesvirus-like genes that are absent in β-herpesviruses and contain 60 novel open reading frames not found in any other herpesvirus [12, 14]. These findings suggest that this particular virus is so different from other herpesviruses that it may be considered as a new herpesvirus subfamily [12, 13].

EEHV poses a threat to the conservation mission of zoos. Several studies have shown that DNAemia can be detected in Asian elephants before the onset of clinical signs and in elephants that do not display clinical signs using PCR techniques [15–19]. To date, this is the only option to monitor a complete herd, and only allows identification of animals with an active infection. Therefore, the detection of antibodies against EEHV likely offers a better strategy to determine which animals are carriers of EEHV. Ideally, antibody detection against the whole virus is preferable, but since the virus cannot be cultured *in vitro*, the best alternative is to use an immunodominant protein, such as glycoprotein B (gB), as an antigen. The viral envelope protein glycoprotein B of herpesviruses is involved in host cell entry and represents an important target of neutralizing antibodies in the host [20, 21]. In this study a serologic test based on the EEHV-gB antigen is described. This assay was used to assess seroprevalence against EEHV-gB in serum and heparin plasma samples from two large cohorts of European and North-American captive Asian elephants. These data are useful to link seroprevalence with EEHV virus detection by PCR methods and, at the end, may give better insight into EEHV dynamics within elephant populations.

## Methods

### Cloning, expression and large scale production of glycoprotein B

The viral sequence of EEHV-1A glycoprotein B [GenBank: AF411189] [22] comprising the start codon up to the transmembrane domain was synthesized (BaseClear, The Netherlands) and cloned, using XhoI/PstI restriction sites, into the multiple cloning site in frame with a C-terminal myc/^6^His tag, but in an alternative open reading frame (ORF) relative to the Shine Dalgarno ribosomal binding site, of the bacterial expression vector pTrcHis2A (Invitrogen). Induction of recombinant glycoprotein B expression was performed in *E.coli* Rosetta2(DE3)pLysS codon plus strain (Millipore). This strain supplies tRNAs for 7 rare codons (AGA, AGG, AUA, CUA, GGA, CCC, and CGG) in order to improve the expression of heterologous proteins.

A single colony transformed with the pTrcHis2A-gBmyc/his plasmid was inoculated into fresh LB medium containing 100 μg/ml ampicillin and 50 μg/ml chloramphenicol and grown overnight at 37 °C in a shaker incubator. The next day 2 l fresh LB medium without antibiotics was inoculated 1 in 100 with the pre-culture and grown in a shaker incubator at 37 °C to an OD_600_ ~ 0.6. The recombinant protein expression was induced by adding IPTG to an end concentration of 1 mM. The temperature was lowered to 25 °C for optimal recombinant protein expression.

Cells were harvested 4 h after induction and pelleted at 6000xG for 10 min in a Beckman Highspeed centrifuge using the JLA16.250 rotor. The supernatant was decanted and the cells were resuspended in native lysisbuffer (500 mM of NaCl, 50 mM phosphatebuffer (pH 8.0), 5 % v/v glycerol and 10 mM of Imidazole and protease inhibitors). Lysozyme was added to an end concentration of 1 mM and the cells were treated 30 min at 4 °C at continuous agitation. The cells were cracked by three freeze/thaw cycles and sonification with a microtip; 8 x 30s at 60 % on ice (Sonopulse HD2070, Bandelin, Germany). The lysate was cleared by ultracentrifugation for 2 h at 27.000 rpm in a Beckman Optima L-90 K ultracentrifuge using the SW32Ti rotor. A similar procedure was conducted with another pTrcHis2A vector encoding a 27 kD irrelevant ^6^His-tagged protein that was used as a negative control.

### Purification of glycoprotein B

The cleared lysate was loaded onto Ni-NTA resin (Superflow, IBA) which was preconditioned with water and 1x native lysisbuffer. Binding was performed overnight at +4 °C under continuous agitation. Next day, the resin was settled vertically and washed with 6 bed volumes of lysisbuffer containing 20 mM of Imidazole. Thereafter, the bound recombinant protein was eluted with 4 bed volumes of elution buffer (500 mM of NaCl, 50 mM phosphate buffer (pH 8.0), 5 % v/v glycerol and 300 mM of Imidazole). Subsequently, salts were removed by dialysis in a Slide-a-Lyzer cassette (3,500 MWCO, 3–12 ml capacity, ThermoScientific) for 48 h at +4 °C. The dialysis buffer (PBS complemented with 0.1 M NaCl and 5 % v/v glycerol) was refreshed three times. After dialysis the recombinant protein content was analyzed for protein concentration (BCA assay, ThermoScientific) and Western Blot. A second round of purification was performed by fast protein liquid chromatography (FPLC) with a 1 ml volume HisTrap-HP^TM^ column (GE Health Care life sciences) with a pressure flow of 0.15 ml/min. The his-tagged proteins were eluted from the column with 10 column volumes (CV) of a linear gradient ranging from 50 mM Imidazole up to 500 mM Imidazole in 0.5 ml aliquots. These were analyzed by Western blot and the eluates containing the his-tagged proteins were subsequently pooled and dialyzed as described above.

### Rabbit anti-EEHV glycoprotein B serum

Five peptide sequences (QDLTVTVSTKKKTF, YNGQNNKKFSEPSTK, VLDTDSDKKNYSYMS, ANVTSRRRKRDANTA and EPSTKFKVYKDYERLQ) derived from the amino acid sequence of glycoprotein B of EEHV1A [GenBank: AF411189] were synthesized and coupled to LPH (Limulus polyphemus hemocyanin) carrier protein. Ten rabbits (*n* = 2 Zimmerman rabbits/peptide) were immunized six times intramuscularly with 10 μg of the respective peptides. Final bleeding was at day 133 after the first vaccination. The polyclonal rabbit sera were produced by Biogenes GmbH (Germany) according to European law on animal welfare. All rabbit sera were highly reactive against the respective peptide as tested in a peptide ELISA assay.

### Immunodetection of recombinant glycoprotein B

Recombinant glycoprotein B antigen was analyzed by separation of small samples from the purification process on a 10 % SDS-PAGE gel (BioRad Laboratories) and separated products were subsequently transferred to PVDF membrane (Immobilon, Millipore). Gels were run in duplicate; one was Coomassie Blue stained for total protein and the duplicate was analyzed with Western blot using an anti-^6^His tag mouse monoclonal antibody (Clontech). As a secondary antibody goat anti-mouse IRdye680 (Licor) was used.

Rabbit serum reactivity with recombinant glycoprotein B was detected with Western Blot by incubating the membranes with rabbit serum (1:400 dilution) followed by detection with goat-anti-rabbit IRdye680 (Licor). Similar strategy was done for elephant serum (1:1000 dilution), but primary antibody was detected with rabbit-anti Asian elephant IgG (1:1000 dilution). Sera of rabbits as well as elephants were pre-incubated in buffer containing *E.coli* lysate (~3 μg/ml) for 1 h at room temperature in order to reduce possible *E.coli* reactivity. The signal of the conjugate was detected with the Licor Odyssey scanner (Licor).

### Glycoprotein B specific capture ELISA

To avoid non-specific reaction against *E.coli* contaminants which co-purified with the ^6^His-tagged EEHV gB antigen during Ni-NTA purification an optimal concentration of 100 ng/well of mouse-anti ^6^His antibody (ClonTech) as a capture antibody was coated in high binding 96-well microtiterplate (Costar). The microtiter plates were blocked with 100 μl/well of PBS containing 2 % w/v BSA in PBS for one hour at 37 °C and washed. All washing steps were performed three times with 200 μl of wash buffer (PBS + 0.05 % v/v Tween20). To determine the optimal antigen concentration for use in the ELISA, the gB antigen was diluted two-fold starting at 2400 ng per well. A pool of rabbit sera was used as anti-gB positive serum.

The purified EEHV-gB antigen was diluted in PBS containing 0.1 % Triton-X to a concentration of 750 ng/well and bound to the capture antibody for 1 h at 37 °C.

After washing, primary incubation was performed with 100 μl/well diluted (1:100 and 1:200) elephant serum in ELISA buffer containing PBS, 0.2 % BSA, 0.1 % non-fat dried powder milk and 3 % w/v NaCl and incubated for one hour at 37 °C. After washing, elephant specific IgG’s bound to glycoprotein B were detected by incubation with 100 μl/well of rabbit anti-elephant IgG (1:1000 diluted in ELISA buffer) for one hour at 37 °C followed by an incubation step with swine anti-rabbit IgG HRPO conjugate (1:1000 diluted in ELISA buffer). After washing 100 μl/well 1xTMB substrate (KPL laboratories) was added, incubated for 10 min at room temperature. The reaction was stopped by adding 100 μl 0.5 M H_2_SO_4_ per well. The absorbance (OD) was measured at 450 nm with 620 nm reference filter (A_450/620_) on a 96-well plate spectrophotometer (Tecan).

### Study cohort in Europe/USA

The elephants in this study were kept in zoos as part of the European Endangered Species Program (EEP, Europe) and the Species Survival Program (SSP,USA). Within these programs elephants are trained to allow voluntarily blood collection for several reasons: monitoring health parameters, monitoring reproduction parameters and providing (retrospective) data for new diagnostic technologies for diseases that may threaten their health. The use of banked elephant blood samples for a retrospective research study falls within the law. All participating zoos reviewed and approved the research proposal for this study and allowed the use of their banked elephant blood samples.

All elephants in the study were Asian elephants; Table 1 (Asian elephants located in Europe) and 2 (Asian elephants located in North America) summarize the age, sex, and clinical status of elephants which will be discussed in detail in this study. All participating zoos reviewed and approved the research described in this study. Initially, 831 serum or heparin plasma samples of 125 Asian elephants located in 14 European zoos were tested for validation of the capture ELISA test. The cut-off value was calculated as a mean OD of 37 samples of 19 animals which scored consistently on the base line level of the test.

**Table 1:** Age, sex, and clinical status of elephants in the study cohort of European zoos

Zoo	Animal	Sex	Born	Status	Age	Serostatus	Sample #	Period	PCR detection	Detection site	Clinical signs	Remark
1	A	F	captive	alive	44	pos	>50	2002–2014	EEHV1B - 2007/2008	O/V	subclinical	ref [17]
1	A1	F	captive	alive	14	pos	>50	2002–2014	EEHV1B - 2007	O	subclinical	ref [17]
1	C	F	wild	alive	28	border	>50	2002–2012	EEHV1B - 2008	O	subclinical	ref [17]
1	D	F	captive	alive	12	ND	>50	2013–2014	EEHV1B - 2008	O	subclinical	ref [17]
1	E	M	captive	alive	17	pos	2	2013–2014	EEHV1 - 2014	TW/C	subclinical	
2	A	M	captive	alive	23	ND	>50	2006–2013	not detected			
2	B	F	wild	alive	31	border	>50	2006–2013	EEHV5 -2011	TW	subclinical	
2	B1	M	captive	alive	10	pos	40–50	2006–2011	EEHV1A - 2008/2011	WB	subclinical	
2	B2	M	captive	dead	1	pos	3	2006–2013	EEHV1A - 2009	necropsy	fatality	
2	B3	M	captive	alive	3	pos	8	2012–2013	EEHV1 - 2013	WB	clinically ill 2013	
2	C	F	wild	alive	33	ND	40–50	2006–2013	EEHV1A - 2011	TW	subclinical	negative study control
2	D	F	wild	alive	33	border	40–50	2006–2013	not detected	nd	healthy	
2	E	F	captive	alive	32	pos	>50	2006–2013	EEHV1A - 2011	TW	subclinical	
2	E1	F	captive	dead	2	pos	3	2006	EEHV1B - 2006	necropsy	fatality	ref [14]
2	E2	F	captive	dead	2	pos	3	2006–2013	EEHV1A - 2009	necropsy	fatality	
2	E3	F	captive	alive	5	pos	>50	2006–2013	EEHV1A - 2011	TW	subclinical	
2	F	F	captive	alive	16	ND	>50	2006–2013	EEHV1A - 2011	U/V	subclinical	
2	F1	M	captive	alive	5	pos	20	2011–2013	EEHV1A - 2011	TW	subclinical	
3	A	F	captive	alive	19	ND	40–50	2007–2012	not available			
3	B	F	wild	alive	30	pos	40–50	2006–2012	not available			
3	C	F	wild	alive	30	border	20	2012	not available			
3	C1	F	captive	alive	16	border	20	2012	not available			
4	A	M	captive	alive	9	pos	2	2007–2014	not done			positive study control
5	A	F	wild	alive	29	pos	1	2002	not done			positive study control

Zoos are numbered, elephants are indicated with a letter and subsequent numbers describe maternal offspring

*ND*, not detectable

*TW*, trunk wash

*C*, conjunctiva

*U*, urine

*O*, oral lesion

*WB*, whole blood

*V*, vulva

Three European zoos (Zoo 1, 2 and 3) provided Asian elephant serum or plasma samples for a longitudinal seroprevalence study ranging from 250 up to 600 days for multiple animals in their herds. Moreover, the samples from Zoo 1 and 2 (including trunkwash and conjunctiva swabs) were also analyzed for EEHV presence by qPCR.

In the USA cohort 476 Asian elephant samples were tested from 63 animals in 17 zoos in the period 2006 until 2012 with a six month interval.

All samples were tested in 1:100 and 1:200 dilutions and the signal to background OD was calculated for both dilutions. A sample was considered positive when both dilutions produced ODs 3 times of the background OD and undetectable when both dilutions produced less than 2 times the background OD. A sample was considered as borderline when one or both dilutions measured an OD between 2–3 times the background OD values.

An animal was considered to be seropositive when all analyzed samples scored positive with an occasional borderline score. An animal was considered borderline seropositive when the majority of its samples scored borderline. An animal was considered undetectable when all of its samples were undetectable.

## Results

### Expression and purification of EEHV glycoprotein B

**Fig. 1:**
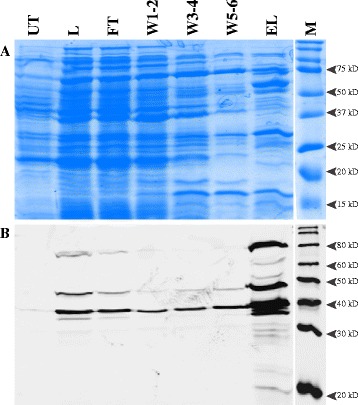
SDS-PAGE total protein analysis (panel **a**) and Western blot analysis with an anti-His monoclonal antibody (panel **b**) demonstrate the purification of full length EEHV-gBHis (~87 kD) and several smaller fragments with a his-tag produced from the EEHV-gBHis open reading frame (lane EL, eluate). During purification a small portion of the recombinant protein is lost in the flow through of the NiNTA column (lane FT) and during the washing steps (lane W1-2, W3-4, W5-6). The empty vector does not yield any his-tagged proteins (lane UT). Lane M is the molecular weight marker

**Fig. 2:**
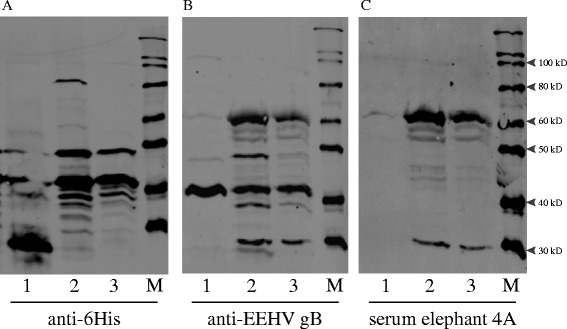
Western blot analysis of an irrelevant 27 kD his-tagged protein and (lane 1), single NiNTA purified EEHV-gBHis (lane 2) and double purified NiNTA/HisTrap FPLC (lane 3) with three different antisera. **a** Western blot with mouse monoclonal anti-His demonstrates the 27 kD His-tagged protein in lane 1 and the highly fragmented EEHV-gBHis in lane 2 and 3. Double purification results in loss of the full length EEHV-gBHis (lane 3). **b**The rabbit peptide (ANVTSRRRKRDANTA) EEHV-gB specific antiserum recognizes non-his tagged bands. **c** Serum of elephant 4A detects identical bands as the rabbit peptide specific serum in the EEHV-gBHis recombinant protein, except for the ~32 kD band which might be an *E.coli* background band (panel **b**)

Positioning of the EEHV-gB open reading frame (ORF) in an alternative position downstream of the Shine Dalgarno ribosomal binding site (RBS) severely reduced the expression levels of the recombinant EEHV-gB, but retained the recombinant protein in the soluble fraction and in its native conformation. Purification of the recombinant EEHV-gB was performed using nickel columns (Fig. 1). Full length EEHV-gB has an expected molecular size of 87 kD, and a band at this expected size was observed (Fig. 1b). Since the portion of recombinant gB protein in the *E.coli* lysate was low many native proteins of *E. coli* Rosetta2 that displayed affinity to nickel in the Immobilized Metal Affinity Chromatography (IMAC) were co-purified during Ni-NTA purification. Full length as well as many other molecular weight sizes, were detected by anti-^6^His-tag antibody detection on Western Blot. Western blot analysis with the EEHV-gB peptide specific rabbit (ANVTSRRRKRDANTA) serum showed more bands with different molecular weights compared to the pattern obtained with anti-his antibody (Fig. 2a and b). Several specific smaller bands were also detected. An elephant serum, which did contain EEHV-gB specific antibodies, as detected with the described sandwich ELISA, did not recognize as many of the EEHV-gB fragments on Western blot (Fig. 2c). Since the double purified (Ni-NTA followed by FPLC Histrap) lost a portion of the full length EEHV-gB (Fig. 2a) the single Ni-NTA purified antigen was further used to develop an ELISA test.

### Validation of an EEHV glycoprotein B specific capture ELISA

**Fig. 3:**
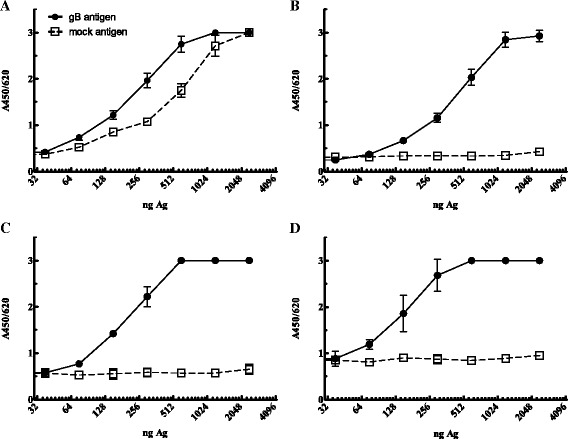
*E. coli* protein contamination in the purified antigen (Ag) induces background signal in an indirect ELISA (panel **a**). This background is reduced in a sandwich ELISA using a mouse monoclonal anti-his antibody as a capture antibody for the target antigen (panel **b**-**d**). Pooled EEHV-gB peptide specific rabbit serum was used in a 1:200 dilution. Panel A no capture antibody, panel B 100 ng capture antibody, panel **c** 200 ng capture antibody, panel D 300 ng capture antibody

**Fig. 4:**
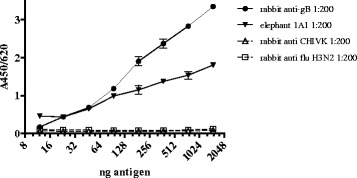
Specificity of the EEHV-gB sandwich ELISA was determined by antigen titration with the EEHV-gB peptide specific rabbit serum, one serum of an EEHV1 PCR positive elephant and two irrelevant rabbit sera. All sera were diluted 1:200 in ELISA buffer

**Fig. 5:**
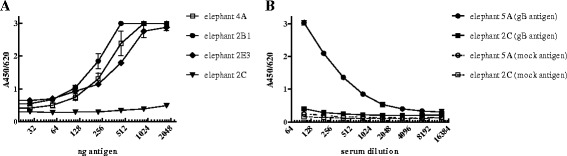
Characterization of the sandwich ELISA with elephant sera. Elephant sera react in a dose-dependent manner as shown by antigen titration (panel **a**) and are specific for the EEHV-gB antigen only (panel **b**)

To reduce aspecific *E.coli* protein reactivity (Fig. 3a) the ELISA assay was setup as a capture ELISA using a mouse-anti ^6^His monoclonal antibody. Pooled sera of rabbits immunized with EEHV-specific peptides were used as a positive control. Figure 3 shows that 100 ng of the capture antibody reduces aspecific reactivity to an acceptable background level of an A_450/620_ of ~0.2. Subsequently, the capture ELISA was further validated with an antigen checkerboard titration to test its specificity with various irrelevant rabbit sera. The results in Fig. 4 demonstrate a dose-related response only when EEHV-gB specific sera were used. Irrelevant rabbit antisera did not react with the antigen. To detect antibody reactivity in the linear range an optimum of 750 ng antigen/well was used in the ELISA, which was used to evaluate the elephant sera cohorts. Once the capture ELISA was developed with the EEHV-gB specific rabbit sera, a large panel of elephant sera was tested to screen for sera with specific reactivity. In total 831 serum or plasma samples of Asian elephants, representing 125 animals of 14 zoos were tested. Twenty-three animals from 7 zoos tested consistently positive (18 %), 24 animals of 5 zoos tested intermittently positive/borderline/undetectable (19 %) and 78 animals tested consistently undetectable (63 %). The test was repeated twice in separate experiments and the results were reproducible. We found three sera with extremely high reactivity in the capture ELISA (elephant 2B1, 4A and 5A respectively, Table 1) and several non-reactive sera (lowest responder was elephant 2C). These elephant sera were used to test again the antigen dose and specificity (Fig. 5). The sera of elephant 2B1, 4A and 5A showed a dose-dependent reactivity whereas the serum from elephant 2C did not (Fig. 5a). Serial dilutions of serum of animal 5A (positive serum) and animal 2C (undetectable serum) on EEHV-gB antigen or an irrelevant ^6^His-tagged protein were used in the assay to test the specificity. Only the positive serum of animal 5A reacted in a dose-dependent manner with EEHV-gB antigen and not with the irrelevant antigen (Fig. 5b).

### The majority of EEHV PCR positive elephants display EEHV specific antibodies

For 36 animals (Table 1 and 2) EEHV PCR data was available, either from published data [12, 14, 15, 17, 19, 23, 24] or personal communications. We compared these results with our ELISA results. Nineteen out of 25 PCR positive animals (76 %) displayed antibodies, of which 16 had significant antibody titers and three had borderline titers. However, in six out of 25 animals no EEHV specific antibodies could be detected (elephants 1D, 2C, 2 F, 6A, 6C, 6C1) while they had been tested positive with the EEHV specific PCR. Eight elephants displayed EEHV-gB specific antibodies, but never tested positive in a routine PCR (elephant 2D, 7A, 7B1, 7D, 7E, 9B1-1, 9C and 9D). Four animals were EEHV5 PCR positive of which three displayed antibodies against EEHV-gB.

**Table 2:** age, sex, and clinical EEHV status of elephants in the study cohort of USA zoos

Zoo	Animal	Sex	Born	Status	Age	Serostatus	Sample #	Period	PCR detection	Detection site	Clinical signs	Remark
1	A	M	wild	alive	50	ND	13	2006–2012	EEHV5A (2011)	TW	subclinical	
1	B	F	wild	alive	46	border	13	2006–2012	EEHV1B (2009)/EEHV5(2011)	WB	Lethargic	ref [23, 24]
1	C	F	wild	alive	34	ND	9	2008–2012	EEHV1A-(2009)/EEHV5B (2011)	TW	subclinical	ref [15]
1	C1	M	captive	alive	10	ND	9	2008–2012	EEHV1A/EEHV1B/EEHV5B	TW/WB	subclinical	ref [15, 23]
1	C2	F	captive	alive	4	pos	2	2012	EEHV5A (2011)	TW	Subclinical	ref [15]
2	D	F	captive	alive	24	pos	13	2006–2012	EEHV1A(2009)/EEHV1B (2010)	TW/WB 2010	Subclinical	ref [12, 19, 24]
2	D1	M	captive	alive	5	pos	2	2010–2012	EEHV5A (2011)	TW/WB	Temporal gland swelling	ref [15]
2	A	F	wild	alive	44	pos	11	2006–2011	not detected			
2	B	F	wild	alive	44	ND	12	2006–2011	not detected			
2	B1	M	captive	alive	22	pos	11	2006–2011	not detected			
2	C	F	wild	alive	44	ND	11	2006–2011	not detected			ref [19]
2	C1	F	captive	alive	8	pos	6	2009–2011	EEHV1A (2010)/EEHV1B (2009)	WB/TW	subclinical feb/dec 2009	ref [19]
2	D	F	wild	alive	35	pos	11	2006–2011	not detected			
2	E	F	captive	alive	18	pos	11	2006–2011	not detected			
2	E1	F	captive	alive	8	pos	5	2009–2011	EEHV1A/EEHV1B (2009)	WB/TW	clinically ill feb/dec 2009	ref [19]
2	A	F	wild	alive	45	pos	7	2006–2010	not available			
2	B	F	wild	alive	44	pos	7	2006–2010	not available			
2	B1	F	captive	alive	16	pos	7	2006–2010	not available			
3	C	F	wild	dead	41	border	4	2006–2007	not available			
3	D	F	captive	alive	26	pos	7	2006–2010	not available			
3	A	F	wild	alive	48	border	10	2006–2011	not done			
3	B	F	wild	alive	41	border	9	2006–2011	not done			
4	B1	F	captive	alive	22	pos	10	2006–2011	not done			
5	B1-1	F	captive	alive	5	pos	1	2010	not detected			
1	C	M	captive	alive	17	pos	10	2006–2011	not detected			
1	D	M	captive	alive	16	pos	7	2006–2009	not detected			

Zoos are numbered, elephants are indicated with a letter and subsequent numbers describe maternal offspring

*ND*, not detectable

*TW*, trunk wash

*C*, conjunctiva

*U*, urine

*O*, oral lesion

*WB*, whole blood

*V*, vulva

### Seroprevalence of a European study cohort

**Fig. 6:**
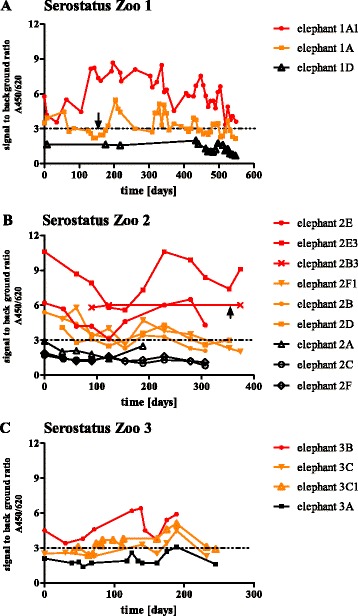
Serostatus of three European zoos measured as the signal to background ratio over a period of time. Zoo 1 (panel **a**) was studied from October 2012 until April 2014. Zoo 2 (panel **b**) was studied from May 2012 until May 2013 and Zoo 3 (panel **c**) from January 2012 until September 2012. The arrow indicates a positive EEHV1 PCR of the trunk wash (animal 1A) or whole blood (animal 2B3). Elephants with non-detectable levels of EEHV antibodies are indicated in black, elephants with an alternating serostatus in orange and elephants with continuous anti-EEHV antibodies in red

Three European zoos were approached to collaborate in our study; their animals are listed in Table 1 (zoo 1 to 3) and their Asian elephant sera collections were analyzed over a substantial period of time (600 days Zoo 1, 400 days Zoo 2 and 250 days Zoo 3). During the study period the animals of Zoo 1 and 2 were screened for EEHV virus presence in whole blood, trunk wash and conjunctiva swabs by either conventional or real time PCR. Figure 6 displays the serostatus of these three herds; the observed patterns showed a rise-and-fall in signal to background ratios (thus, relative antibody quantity). A similar rise-and-fall pattern was observed for animals, which scored below the detection level. Additional clinical evaluations of the animals in this study can be found in (Additional file 1).

Only two animals had a positive PCR in the study-period in either trunk wash (animal 1A) or whole blood (animal 2B3). As seen in Fig. 6a animal 1A was EEHV1 PCR positive in trunk wash on day 150 and, shortly afterwards, had elevated antibody titers up to 8 weeks after the PCR positive trunk wash. As shown in Fig. 6b calf 2B3 developed a DNAemia and clinical signs of EEHV disease on day 370 of the study period with typical EEHV clinical signs. However, this calf was already seropositive on day 90 of the study period. He survived this episode with supportive treatment and famciclovir administration. Eighteen days prior to disease onset his half-sister 2E3 showed a rise in antibody titers. Unfortunately no samples of the other herd members were available in this period of the study.

## Discussion

To date, an accurate and dependable test to evaluate EEHV seroprevalence in Asian elephants has not been developed. In part, this is due to the inability to culture the virus *in vitro* and therefore whole virus is not available as the ideal antigen source for an ELISA. Production of recombinant proteins from one of the EEHV glycoproteins, like gB, has proven to be a major challenge. Here we describe a novel ELISA that specifically detects EEHV-gB antibodies circulating in Asian elephants. We were able to link seroprevalence to PCR detection of the EEHV in approximately 80 % animals. This novel assay will provide more insight into the epidemiology of this hitherto poorly understood virus.

The antigen expression, production and purification methods for fragmented EEHV-gB (genotype 1A) driven by a bacterial Shine Dalgarno promoter in an alternative open reading frame, yielded antigens for a sandwich ELISA specific for EEHV-gB. Incomplete EEHV-gB amino acid sequences were produced from the plasmid presumably due to programmed ribosomal frameshifting (PRF) and alternative translation initiation sites. PRF and alternative translation initiation are well-described phenomena [25–28] and can occur in prokaryotes, as well as in eukaryotes. PRF occurs when the ribosome encounters a so-called hungry codon (low level of acetylated tRNA) in its A-site followed by a slippery sequence in the P-site of the ribosomal complex. In our expression system, it is likely that the Shine Dalgarno sequence RBS initiates the overexpression of a short nonsense peptide of 36 amino acids thereby releasing the ribosomes quickly in order to re-initiate translation with a high likelihood to undergo PRF since this small ORF contains multiple hungry codons. As a side product, fragments of EEHV-gB specific sequences, either with or without a his-tag were made and confirmed by Western blot analysis. This suggested that the non-his-tagged fragments were co-purified during the purification process. EEHV-gB peptide specific rabbit sera were able to detect the antigen fragments, whereas positive elephant sera recognized fewer of these fragmented antigen products. It is likely that the majority of the anti-EEHV-gB antibodies in elephants recognize conformational epitopes, which are linearized in Western Blot. This is supported by the fact that the produced antigen was highly reactive in the sandwich ELISA test with the positive rabbit sera as well as many elephant sera and not with irrelevant sera. As a result, it is likely that this ELISA would fail to detect antibodies directed to those conformational epitopes of gB which do not form properly in the bacterial expression machinery. In this study no seronegative elephants were found. The background of the described assay was set up with sera of elephants, which were consistently found on the non-detectable range of the assay, but still ~0.1 OD higher than naïve rabbit serum. This test was developed with a capture antibody, which did not completely reduce the background to an OD of 0. Therefore, it is highly likely that the cut-off value was leveled unrealistically high. In the study cohort we found several seropositive elephants, which were routinely diagnosed with other subtypes than EEHV1A by PCR. The obtained data suggest that this test may be able to detect antibodies against gB from EEHV subtypes other than EEHV1A; at least EEHV1B and EEHV5. Divergence on the amino acid level of the glycoprotein B of the subtypes EEHV ranges from 11 % (EEHV1A vs EEHV1B) up to 21 % (EEHV1A vs EEHV5) [23]. However, we cannot exclude that these animals did had an EEHV1A co-infection as well. It is interesting to speculate that genetic background plays a role in susceptibility to develop severe disease. However, better designed study cohorts, which include serology as well as PCR detection of the virus are required in order to address the question whether the host genetic background has a major role in fatal EEHV disease.

## Conclusion

We have shown that it is possible to express the gB of EEHV in a bacterial system by using alternative translation upstream of the Shine Dalgarno sequence. Further, our results and those of recent reports [15, 17, 19, 29] demonstrate that EEHV prevalence in captive born as well as wild-born elephants (as measured by virus detection with PCR techniques in whole blood and trunk secretions) is much higher than initially anticipated. These studies underline that elephants may be the natural host of EEHV that, in general, does not cause major disease. The methodology and data presented in this article give a novel insight into the EEHV1 seroprevalence in Asian elephants. As expected, a large portion of elephants showed antibodies against EEHV, but not all. Additionally, the serostatus of elephants within a herd may vary from non-detectable up to high titers.

## Additional file

Additional file 1:
**Supplementary information.** (DOCX 125 kb)

## Abbreviations

BCA: Bicinchoninic acid
BSA: Bovine serum albumin
CV: Column volume
EEHV: Elephant endotheliotropic herpes virus
EEP: Endangered species program
ELISA: Enzyme linked immunosorbent assay
EM: *Elephas maximus*
FPLC: Fast protein liquid chromatography
gB: Glycoprotein B
HPRO: Horse radish peroxidase
IMAC: Immobilized metal affinity chromatography
IPTG: Isopropyl β-D-thiogalactoside
LA: *Loxodonta africana*
LB: Luria Bertani broth
LPH: Limulus polyphemus hemocyanin
Ni-NTA: Nickel nitrilotriacetic acid
nm: Nanometer
OD: Optical density
ORF: Open reading frame
PBS: Phosphate buffered saline
PCR: Polymerase chain reaction
PRF: Programmed ribosomal frameshifting
PVDF: Polyvinylidene difluoride
qPCR: Quantative polymerase chain reaction
RBS: Ribosomal binding site
SDS-PAGE: Sodium dodecyl sulphate polyacrylamide gel electrophoresis
SSP: Species survival program
TMB: 3,3’,5,5’-tetramethylbenzidine
BCA: Bicinchoninic acid
BSA: Bovine serum albumin
CV: Column volume
EEHV: Elephant endotheliotropic herpes virus
ELISA: Enzyme linked immunosorbent assay
EM: *Elephas maximus*
FPLC: Fast protein liquid chromatography
gB: Glycoprotein B
HPRO: Horseradish peroxidase
IMAC: Immobilized metal affinity chromatography
IPTG: Isopropyl b-D-thiogalactoside
LA: *Loxodonta africana*
LB: Luria Bertani broth
LPH: Limulus polyphemus hemocyanin
Ni-NTA: Nickel nitrilotriacetic acid
nm: Nanometer
OD: Optical density
ORF: Open reading frame
PBS: Phosphate buffered saline
PCR: Polymerase chain reactionPRF, programmed ribosomal frameshifting
PVDF: Polyvinylidene difluoride
qPCR: Quantative polymerase chain reaction
RBS: Ribosomal binding site
SDS-PAGE: Sodium dodecyl sulphate polyacrylamide gel electrophoresis
TMB: 3,3’,5,5’-tetramethylbenzidine
