# Erratum to: A novel antigen capture ELISA for the specific detection of IgG antibodies to elephant endotheliotropic herpes virus

**DOI:** 10.1186/s12889-015-2178-3

**Published:** 2015-09-03

**Authors:** Petra B. van den Doel, Víctor Rodríguez Prieto, Sarah E. van Rossum-Fikkert, Willem Schaftenaar, Erin Latimer, Lauren Howard, Sarah Chapman, Nic Masters, Albert D. M. E. Osterhaus, Paul D. Ling, Akbar Dastjerdi, Byron Martina

**Affiliations:** ViroScience Lab, Erasmus Medical Center, Erasmus MC, Room Ee1714, dr. Molewaterplein 50, Rotterdam, 3015 GE The Netherlands; Artemis One Health Research Institute, Utrecht, The Netherlands; VISAVET Centre, Animal Health Department, Complutense University of Madrid, Madrid, Spain; Department of Genetics, Erasmus Medical Center, Rotterdam, The Netherlands; Veterinary Services, Rotterdam Zoo, Rotterdam, The Netherlands; Smithsonian Conservation Biology Institute, Smithsonian’s National Zoo, Washington, DC USA; Department of Animal Health, Houston Zoo, Inc., Houston, TX USA; East-Midland Zoological Society, Twycross Zoo, Warwickshire, UK; Veterinary Services, Zoological Society of London, London, UK; Department of Molecular Virology and Microbiology, Baylor’s College of Medicine, Houston, TX USA; Animal and Plant Health Agency, Addlestone, UK

Unfortunately, the original version of this article [1] contained an error. The zoo numbers in Table 2 are incorrect. The correct version of Table 2 can be found below;

**Table 2:** Age, sex, and clinical EEHV status of elephants in the study cohort of USA zoos

Zoo	Animal	Sex	Born	Status	Age	Serostatus	Sample #	Period	PCR detection	Detection site	Clinical signs	Remark
6	A	M	Wild	Alive	50	ND	13	2006-2012	EEHV5A (2011)	TW	Subclinical	
6	B	F	Wild	Alive	46	Border	13	2006-2012	EEHV1B (2009)/EEHV5(2011)	WB	Lethargic	Ref [23, 24]
6	C	F	Wild	Alive	34	ND	9	2008-2012	EEHV1A-(2009)/EEHV5B (2011)	TW	Subclinical	Ref [15]
6	C1	M	Captive	Alive	10	ND	9	2008-2012	EEHV1A/EEHV1B/EEHV5B	TW/WB	subclinical	Ref [15, 23]
6	C2	F	Captive	Alive	4	Pos	2	2012	EEHV5A (2011)	TW	Subclinical	Ref [15]
6	D	F	Captive	Alive	24	Pos	13	2006-2012	EEHV1A(2009)/EEHV1B (2010)	TW/WB 2010	Subclinical	Ref [12, 19, 24]
6	D1	M	Captive	Alive	5	Pos	2	2010-2012	EEHV5A (2011)	TW/WB	Temporal gland swelling	Ref [15]
7	A	F	Wild	Alive	44	Pos	11	2006-2011	Not detected			
7	B	F	Wild	Alive	44	ND	12	2006-2011	Not detected			
7	B1	M	Captive	Alive	22	Pos	11	2006-2011	Not detected			
7	C	F	Wild	Alive	44	ND	11	2006-2011	Not detected			Ref [19]
7	C1	F	Captive	Alive	8	Pos	6	2009-2011	EEHV1A (2010)/EEHV1B (2009)	WB/TW	Subclinical feb/dec 2009	Ref [19]
7	D	F	Wild	Alive	35	Pos	11	2006-2011	Not detected			
7	E	F	Captive	Alive	18	Pos	11	2006-2011	Not detected			
7	E1	F	Captive	Alive	8	Pos	5	2009-2011	EEHV1A/EEHV1B (2009)	WB/TW	Clinically ill feb/dec 2009	Ref [19]
8	A	F	Wild	Alive	45	Pos	7	2006-2010	Not available			
8	B	F	Wild	Alive	44	Pos	7	2006-2010	Not available			
8	B1	F	Captive	Alive	16	Pos	7	2006-2010	Not available			
8	C	F	Wild	Dead	41	Border	4	2006-2007	Not available			
8	D	F	Captive	Alive	26	Pos	7	2006-2010	Not available			
9	A	F	Wild	Alive	48	Border	10	2006-2011	Not done			
9	B	F	Wild	Alive	41	Border	9	2006-2011	Not done			
9	B1	F	Captive	Alive	22	Pos	10	2006-2011	Not done			
9	B1-1	F	Captive	Alive	5	Pos	1	2010	Not detected			
9	C	M	Captive	Alive	17	Pos	10	2006-2011	Not detected			
9	D	M	Captive	Alive	16	Pos	7	2006-2009	Not detected			

Zoos are numbered, elephants are indicated with a letter and subsequent numbers describe maternal offspring

ND, not detectable

TW, trunk wash

C, conjunctiva

U, urine

O, oral lesion

WB, whole blood

V, vulva

