# Supplementary material for: A novel antigen capture ELISA for the specific detection of IgG antibodies to elephant endotheliotropic herpes virus
Source: BMC Vet Res. 2015 Aug 14;11:203. doi: 10.1186/s12917-015-0522-6 (PMC4535388; doi:10.1186/s12917-015-0522-6)
Supplement: Additional file 1: — Supplementary information. (DOCX 125 kb) [file 12917_2015_522_MOESM1_ESM.docx]

**Supplementary information**

**Results**

## Clinical evaluation of the European study cohort

Zoo 1 currently consists of three adult cows (animals 1A, 1A1 and animal 1D), two calves (not in table 1) and one breeding bull (1E). Animal 1C was relocated to another European zoo in 2012. Zoo 1 had at least one fatal EEHV case in 1998 (3-yrs old daughter of the matriarch). In 2002 an 11 months old calf which was a second generation relative of the matriarch of this herd (animal 1A) died of the consequences of jejunum rupture (torsio jejuni and incarceration of the jejunum in a large herniation of the mesentericum, probably of traumatic origin). Three days prior to its death and at necropsy this animal was diagnosed EEHV1 PCR positive. Furthermore, zoo 1 reported in 2007 and 2008 non-fatal clinical presentation of EEHV1B in elephants 1A, 1A1, 1C and 1D [[17](#_ENREF_17)]. In our study we find elephants 1A, 1A1 and 1E seropositive (figure 6A, animal 1E not included). Figure 6A shows typical herpes like reactivation pattern which seems to coincide for animal 1A and 1A1. Animal 1A has an EEHV1 PCR positive trunk wash on day 150 of the study period. None of the other samples in this period tested positive for EEHV1 virus detection. Moreover, one month before delivery of a calf in 2010 the titers of animal 1A1 increased (data not shown) and slowly normalized over the course of three months. We found in the initial cohort a seropositive animal which was relocated to another zoo and is a relative of animals 1A (first generation off-spring) and 1A1 (sibling).
Zoo 2 has an intensive screening program on EEHV and their animals are tested regularly by EEHV specific PCR (trunk wash and whole blood). This zoo has a rather large herd and has been extremely affected with fatal EEHV cases; the first case occurred in 2006 when calf 2E1 died at the age of 2y. In 2009 two calves (elephant 2B2 and 2E2) under 2,5y of age were lost within one month. EEHVgB specific antibodies were found in these animals three months prior to their death. Two days before death the titers were declining. Preceding the latter two cases the adolescent male in this herd (elephant 2B1) had an episode of EEHV1 DNAemia as confirmed by PCR in 2008; he lost positive DNAemia in September 2008. In the initial cohort numerous serum samples of this animal were tested highly positive, similar to his half-sister, animal 2E3 in the period 2006 - 2011. Another EEHV incident occurred in May 2013. A 1.5 yrs old male calf (elephant 2B3) showed clinical signs of EEHV and had confirmed DNAemia. He survived this period after an intensive treatment regimen. Retrospective serum samples as from summer 2012 from this animal showed that this animal had already EEHVgB antibodies at the age of eight months old, very early in life. Due to sporadic sampling in suckling calves we were not able to look into antibodies of very young animals.
Zoo 4 has no history of fatal EEHV cases. Elephant 4A is a captive born 9y old healthy male Asian elephant without a history of clinical illness. This elephant was the only seropositive elephant out of a panel of nine elephants which were tested in this facility in October 2007. Interestingly, we found two siblings of animal 4A (elephants 9C and 9D) in the USA cohort (table 2) as being extremely seropositive as well. Elephant 9C and 9D never were in physical contact with elephant 4A. Their only common factor is their sire which was relocated from a North-American zoo to a European zoo in 2000.
Elephant 5A (a 29y old wild born female Asian elephant) was housed in a circus where the first described European fatal case occurred in July 1988. In 1999 she was relocated to zoo 5 where, one month after her arrival, a 2y old male Asian elephant succumbed to EEHV. The sire from this calf lost in total four calves of EEHV. One of his live offspring is located in a North European zoo and this breeding bull has lost three of his offspring of EEHV as well. Due to CITES legislation we were not able to analyze more serum samples in this very interesting elephant family.

## Clinical evaluation a North American cohort of elephant sera

Seven Asian elephants of zoo 6 were monitored in the period 2006-2012 with a 6 months interval. Here we observed a similar pattern as in zoo 2; the patriarch, with non-detectable EEHV antibodies, has multiple off-springs which succumbed to EEHV. All living off-spring are highly seropositive (animal 6C2 and 6D1). Juvenile herd member 6C1 is not an offspring of the patriarch but does carry the EEHV1 virus as detected by PCR [[19](#_ENREF_19)]. However, he has undetectable levels of EEHV specific antibodies. The adult female elephant 6D is also interesting; she lost her first two off-springs to EEHV herself. Moreover, she also lost three siblings to EEHV. Two out of the three still living siblings of animal 6D are highly EEHV seropositive (7B1 and another elephant not listed in table 2). Again, these animals never had any physical contact with each other. Around the birth of calf 6D1 elephant 6D has raised EEHV specific antibody titers.
The vast majority of the zoo 7 herd is highly seropositive except for two adult cows (7B and 7C). Breeding bull 7B1 has the same sire as elephant 6D. This sire lost four of his descendants from EEHV and at least one of his descendants survived a typical EEHV episode. Breeding bull 7B1 is the sire of elephant 7C1 and 7E1; both these adolescent elephants experienced an episode of clinical EEHV illness of which they both recovered [[19](#_ENREF_19)]. Elephant 7E is a half-sister of breeding bull 2A; their common sire lost two of his descendants from EEHV and one survived critical EEHV illness. Breeding bull 2A lost four of his descendants of EEHV.
The herd of zoo 8 consists of EEHV seropositive animals only. Adult female 8B produced offspring (elephant 8B1) with a breeding bull who also had a fatal case offspring (not listed in table 2). Again, we observe very high titers in animal 8B1, a half-sister of the deceased elephant, which died 15 years before animal 8B1 was born.
The elephants of zoo 9 are all seropositive. In this zoo we found two half-brothers of the adolescent male 4A. Again, these animals had no physical contact with animal 4A; the only common link is their sire. Unlike the above described herds, in this blood line no EEHV fatalities were described.
